# The *ZFHX3* GGC Repeat Expansion Underlying Spinocerebellar Ataxia Type 4 has a Common Ancestral Founder

**DOI:** 10.1002/mds.30077

**Published:** 2024-12-05

**Authors:** Zhongbo Chen, Pilar Alvarez Jerez, Claire Anderson, Martin Paucar, Jasmaine Lee, Daniel Nilsson, Hannah Macpherson, Annarita Scardamaglia, Kylie Montgomery, John Hardy, Andrew B. Singleton, Arianna Tucci, Katherine D. Mathews, Ying‐Hui Fu, Martin Engvall, José Laffita‐Mesa, Inger Nennesmo, Anna Wedell, Louis J. Ptáček, Cornelis Blauwendraat, Emil K. Gustavsson, Per Svenningsson, Mina Ryten, Henry Houlden

**Affiliations:** ^1^ Department of Clinical and Movement Neuroscience Queen Square Institute of Neurology, University College London (UCL) London UK; ^2^ The Francis Crick Institute London UK; ^3^ Department of Neurodegenerative Disease Queen Square Institute of Neurology, UCL London UK; ^4^ Center for Alzheimer's and Related Dementias National Institute on Aging and National Institute of Neurological Disorders and Stroke, National Institutes of Health Bethesda Maryland USA; ^5^ Department of Genetics and Genomic Medicine Great Ormond Street Institute of Child Health, UCL London UK; ^6^ NIHR Great Ormond Street Hospital Biomedical Research Centre, UCL London UK; ^7^ Department of Neurology Karolinska University Hospital Stockholm Sweden; ^8^ Department of Clinical Neuroscience Karolinska Institutet Stockholm Sweden; ^9^ Department of Neuromuscular Disease Queen Square Institute of Neurology, UCL London UK; ^10^ Department of Clinical Genetics Karolinska University Hospital Stockholm Sweden; ^11^ Science for Life Laboratory, Department of Molecular Medicine and Surgery Karolinska Institutet Stockholm Sweden; ^12^ Reta Lila Weston Institute, Queen Square Institute of Neurology, UCL London UK; ^13^ UK Dementia Research Institute, UCL London UK; ^14^ NIHR University College London Hospitals Biomedical Research Centre London UK; ^15^ Institute for Advanced Study The Hong Kong University of Science and Technology Hong Kong China; ^16^ Laboratory of Neurogenetics National Institute on Aging, National Institutes of Health Bethesda Maryland USA; ^17^ William Harvey Research Institute Queen Mary University of London London UK; ^18^ Department of Pediatrics University of Iowa Carver College of Medicine Iowa City Iowa USA; ^19^ Department of Neurology University of Iowa Carver College of Medicine Iowa City Iowa USA; ^20^ Department of Neurology University of California San Francisco San Francisco California USA; ^21^ Institute for Human Genetics University of California San Francisco San Francisco California USA; ^22^ Weill Institute for Neuroscience University of California San Francisco San Francisco California USA; ^23^ Kavli Institute for Fundamental Neuroscience University of California San Francisco San Francisco California USA; ^24^ Department of Molecular Medicine and Surgery Karolinska Institutet Stockholm Sweden; ^25^ Centre for Inherited Metabolic Diseases Karolinska University Hospital Stockholm Sweden; ^26^ Department of Neurobiology Care Sciences and Society, Karolinska Institutet Stockholm Sweden; ^27^ Department of Oncology‐Pathology Karolinska Institutet Stockholm Sweden; ^28^ UK Dementia Research Institute at the University of Cambridge Cambridge UK; ^29^ Department of Clinical Neurosciences School of Clinical Medicine, University of Cambridge Cambridge UK

**Keywords:** ataxia, long‐read sequencing, repeat expansion disorder, haplotype, spinocerebellar ataxia type 4

## Abstract

**Background:**

The identification of a heterozygous exonic GGC repeat expansion in *ZFHX3* underlying spinocerebellar ataxia type 4 (SCA4) has solved a 25‐year diagnostic conundrum. We used adaptive long‐read sequencing to decipher the pathogenic expansion in the index Utah family and an unrelated family from Iowa of Swedish ancestry. Contemporaneous to our discovery, other groups identified the same repeat expansion in affected individuals from Utah, Sweden, and Germany, highlighting the current pivotal time for detection of novel repeat expansion disorders.

**Methods:**

Given that the pathogenic repeat expansion is rare on a population level, we proposed a common ancestor across all families. Here, we employed targeted long‐read sequencing through adaptive sampling, enriching for the chr16q22 region of interest.

**Results:**

Using phased sequencing results from individuals from Utah, Iowa, and Southern Sweden, we confirmed a common ~2000‐year‐old ancestral haplotype harbouring the repeat expansion.

**Conclusion:**

This study provides further insight into the genetic architecture of SCA4. © 2024 The Author(s). *Movement Disorders* published by Wiley Periodicals LLC on behalf of International Parkinson and Movement Disorder Society.

## 1 Introduction

The genetic cause of spinocerebellar ataxia type 4 (SCA4), a late‐onset neurodegenerative condition characterized by cerebellar ataxia and peripheral neuropathy, had evaded diagnosis for more than 25 years despite its clear linkage to chromosome 16q22 defined within a large family of Swedish ancestry residing in Utah (USA).^1^ Advances in long‐read sequencing technologies, bioinformatic tools, and an improved understanding of population‐level structural variation enabled us to recently decipher the causative heterozygous exonic GGC repeat expansion in *ZFHX3* in the index Utah family and an unrelated family from Iowa of Swedish ancestry.^2^ Our work harnessed targeted long‐read sequencing to detect novel repeat expansions in a variant‐agnostic manner. This discovery epitomized both the difficulties and the emerging possibilities for diagnosis of such challenging neurogenetic disorders, which collectively carry a significant disease burden.

Parallel to our work, others have also independently uncovered the causative expansion in individuals with SCA4 from eight separate kindreds from Southern Sweden,^3^, ^4^ Germany, and related individuals to our cohort from Utah.^5^ These individuals with SCA4 presented with variable phenotypes and repeat sizes, culminating in a recent study that showed inverse correlation between repeat length and age‐of‐onset in two German families.^6^ These contemporaneous findings of the GGC repeat expansion across all groups reflects the current pivotal time for novel repeat expansion discovery.^7^, ^8^

It has been proposed that a founder event underlies the repeat expansion haplotype in individuals from Utah and Germany.^5^ We propose that a common Swedish ancestral haplotype containing the repeat expansion is common to all families with SCA4, which has important implications for understanding the genetic architecture of disease.

Here, we were able to assess the detailed phased haplotype structure using long‐read sequencing. We compared the repeat‐containing haplotype of individuals with SCA4 from the index Utah family,^1^ the affected family from Iowa,^2^ and affected individuals from two families hailing from Southern Sweden,^3^ compared with the families from Germany^5^ to show that the repeat expansion‐containing haplotype arose from a common founder event.

## 2 Methods

### 2.1 Patients and Participants

This study was approved by the Institutional Review Board for Human Research at the University of Utah School of Medicine with written informed consent obtained from each participant. The Swedish Ethical Review Authority (Etikprövningsnämnden dnr 2010/1659) also approved this study with written informed consent from each participant.

### 2.2 DNA Extraction

Methods for DNA extraction and preparation from Utah and Iowa families were as previously described.^2^ Briefly, lymphoblast cell lines of participants from the Utah SCA4 family were cultured. Genomic DNA (gDNA) was extracted from 1 × 10^6^ harvested cells using the New England Biolabs Monarch High Molecular Weight DNA Extraction Kit for Cells and Blood standard protocol. High molecular weight (HMW) DNA from lymphoblasts were pre‐sheared or sheared as necessary to 12–17 kb. Blood‐derived gDNA from the Iowa individuals ranged in size from 8 to 17 kb and did not require shearing.^2^

For the individuals affected by SCA4 from Sweden, HMW genomic DNA was extracted from 18–25 mg of brain tissue using the Monarch HMW DNA Extraction Kit for Tissue kit following the standard input protocol. Tissue chips from the thalamus and hypothalamus for patient Sweden1 and thalamus and cerebellum for patient Sweden2 were manually homogenized on ice using a pestle. Homogenized lysates were incubated in HMW gDNA Tissue Lysis Buffer containing Proteinase K at 56°C for 15 min with agitation at 1400 rpm, then for a further 30 min without agitation. Genomic DNA was eluted in 50 μL. gDNA (3 μg) at a concentration of 15 ng/μL was sheared to 15–17 kb using the Diagenode Megarupter 3 Shearing Kit at speed 30.

Size and quality of genomic DNA from lymphoblasts, blood, and brain tissue were checked using the Genomic DNA 165 kb kit for the Agilent Femto Pulse, NanoDrop, and Qubit.

### 2.3 Targeted Long‐Read DNA Sequencing Using Adaptive Sampling

Swedish brain tissue‐derived gDNA underwent a 1X Promega ProNex bead cleanup before 1.3–2.2 μg was inputted into the ONT SQK‐NBD114.24 native barcoding (Oxford, UK) protocol as previously described^2^ (https://www.protocols.io/view/native-barcoding-sqk-nbd114-gdna-for-adaptive-samp-kxygx3qx4g8j/v1).^9^ Equimolar quantities of the barcoded samples were pooled into three libraries of 50 fmol each, loaded onto one R10.4.1 flow cell and sequenced using PromethION. We used a targeted long‐read sequencing approach through adaptive sampling to enrich sequencing of DNA based on a target sequence, controlled computationally in real time. The library was sequenced using adaptive sampling through a custom browser extensible data (BED) file covering the 20 Mb region of interest (chr16:56,000,000–76,100,000 (hg38)).

### 2.4 Structural Variant and Single Nucleotide Variant Detection and Phasing

High accuracy base calling was performed with Guppy (v.7.0.9, ONT). Resulting FASTQ files, with high accuracy reads (quality score >9), were mapped to the human genome GRCh38 using minimap2 (v.2.26; RRID:SCR_018550). We applied PEPPER‐Margin‐DeepVariant, a haplotype‐aware variant calling pipeline to derive phased BAMs and phased single nucleotide variant (SNV) calls.^10^ We also ran Sniffles2.2.3 on the phased BAM from PEPPER‐Margin‐DeepVariant with the *phase* tag to obtain phased structural variant calls.^11^

### 2.5 Methylation Analysis

We used modbamtools^12^ v.0.4.8 to create haplotype methylation plots for each individual around the repeat site. The detailed protocol has previously been described in https://www.protocols.io/view/processing‐frozen‐cells‐for‐population‐scale‐sqk‐l‐6qpvr347bvmk/v1. In order to compare methylation patterns with unaffected individuals, we used DNA derived from the HG002 cell line from Coriell (https://www.coriell.org/) as previously described as a control.^13^

### 2.6 Haplotyping Analysis

We reviewed phased SNVs called from all individuals for assessment of the presence of rare variants around the repeat expansion. In order to compare the haplotypes in the pathogenic repeat expansion‐containing allele compared with the other allele, we took SNVs present within the repeat‐containing haplotype of the Utah1 individual as the index haplotype for consistency. We then compared whether phased SNVs in other individuals matched the SNVs within the index repeat containing haplotype. Dating of the repeat‐containing haplotype was carried out using previously established methods for rare mutations amongst small sample sets with dense marker data (https://github.com/bahlolab/DatingRareMutations).^14^ The model assumes a correlated genealogy as subsets of individuals likely have common ancestry before the most recent common ancestor as individuals rarely have independent recombination histories.^14^

## 3 Results

Here, we assessed the haplotype structure of individuals with SCA4 from Utah,^1^, ^2^ Iowa,^2^ and Sweden^3^ to ascertain whether the repeat expansion‐containing haplotype arose from a common founder event. Within the two individuals of Swedish ancestry, DNA was extracted from post‐mortem brain samples for analyses, while DNA was extracted from lymphoblastoid cell lines and whole blood for the Utah and Iowa families, respectively.^2^, ^3^ Full demographic information is presented in Table 1. We then leveraged the utility of adaptive sampling to computationally drive targeted long‐read sequencing using Oxford Nanopore Technologies (ONT).^2^, ^9^ This had been an approach we originally employed to enrich for sequencing over the region of high linkage leading to the identification of the *ZFHX3* repeat expansion.^2^, ^9^ Furthermore, this method also enabled haplotypes to be phased reliably. Unfortunately, we were unable to sequence all individuals affected due to lack of adequate quality DNA from historical samples.^2^

**TABLE 1:** Summary of individuals studied

Patient	Case number	Family	Estimated GGC repeats (n)	GGC repeats on other allele (n)	Age‐of‐onset (years)	Sex	Clinical presentation
Utah1	III.2	Utah	51	21	Unknown	F	Loss of balance and peripheral sensory neuropathy
Utah2	IV.4	Utah	51	21	59	M	Loss of balance and peripheral sensory neuropathy
Iowa2	II.1	Iowa	48	21	50	M	Loss of balance and peripheral sensory neuropathy
Sweden1	Family 1 (IV:4)	Sweden	55	21	42	M	Loss of balance, neuropathy, and dysautonomia
Sweden2	Family 2 (III:2)	Sweden	46	21	35	F	Loss of balance and neuropathy

Further information on individuals previously evaluated in studies.^2^, ^3^ For individuals from Sweden (Sweden1 and Sweden2), analysis within this study came from DNA extracted from post‐mortem brain hypothalamus samples, and not lymphocytes, as used in the original study.^3^ Repeat sizes were estimated from Sniffles tool.

Abbreviations: F, female; M, male.

**FIG. 1:**
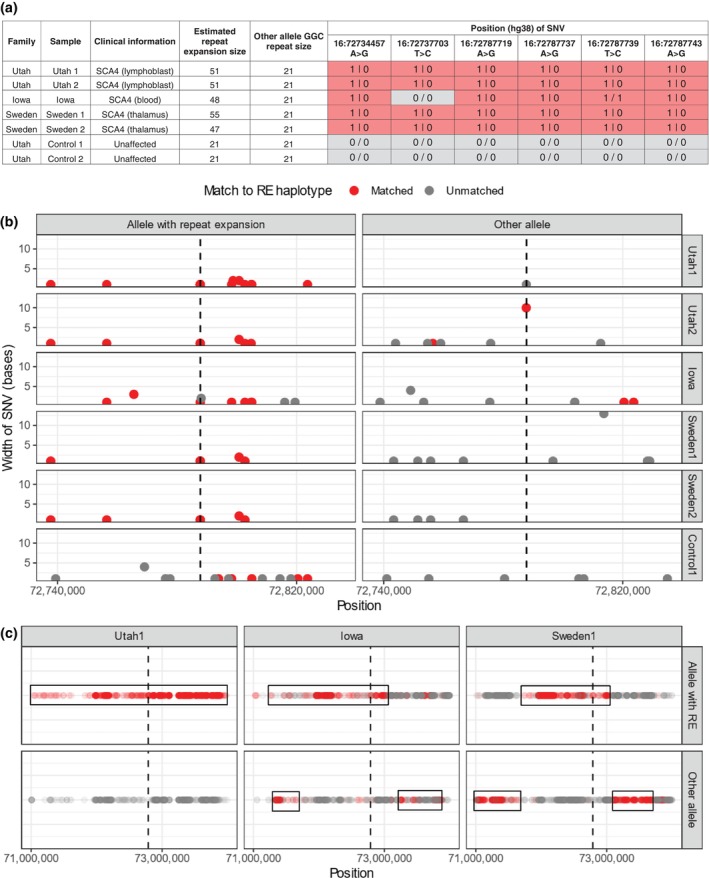
Characterizing the repeat expansion (RE) haplotype using adaptive sampling‐enabled targeted long‐read sequencing. (A) Six ultra‐rare single nucleotide variants (SNVs) identified by Figueroa et al.^5^ were associated with the repeat expansion haplotype in affected individuals from Utah, Iowa, and Sweden. Sizing of the phased structural variants including the GGC repeat expansion used Sniffles.^11^ 1 represents presence of a variant, 0 represents absence of the particular variant. | denotes a phased variant. / denotes unphased variant. The first allele contains the repeat expansion (ie, 1|0 shows the presence of the SNV on the repeat‐expansion‐containing allele. It is worth noting that four SNVs chr16:72787719 A>G; chr16:72787737 A>G; chr16:72787739 T>C; and chr16:72787743 A>G) are located within the expanded repeat (B). SNVs in the repeat expansion haplotype (taking Utah1 repeat expansion haplotype as the index haplotype) within 50 kb of the repeat expansion locus (vertical dashed line) showed strong similarities among cases from Utah, Iowa, and Southern Sweden. SNVs matching the index repeat expansion‐haplotype are shown in red. SNVs present that do not match those within the repeat‐containing haplotype are shown in gray. (C). Representative extended phased haplotypes showed a large identical haplotype block (boxed) with crossover events from individuals with spinocerebellar ataxia type 4 from Utah, Iowa, and Sweden. This led us to conclude that the haplotype associated with *ZFHX3* GGC repeat expansion underlying spinocerebellar ataxia type 4 has a common ancestral founder. [Color figure can be viewed at wileyonlinelibrary.com]

First, we estimated the *ZFHX3* GGC repeat expansion size using Sniffles2 calling from the long‐read sequencing data (Table 1). We then reviewed phased SNVs situated around the repeat expansion. We found that all six of the rare SNVs described in individuals with SCA4 reported by Figueroa and colleagues^5^ were present across individuals from our four families, but only within the allele containing the repeat expansion (Fig. 1A). The only exception was the chr16:72737704T>C, which was absent in one individual from Iowa. Furthermore, these variants were absent in unaffected and unrelated spouses within the Utah kindred.

For consistency,^5^ taking the repeat expansion haplotype in an individual from Utah as the index haplotype, we looked within ±50 kb of the repeat expansion and found high SNV similarity within the repeat expansion‐containing haplotype across all individuals (Fig. 1B). This pattern was not appreciable within unaffected individuals or on the alternate allele (Fig. 1B). Lastly, extended phased haplotypes from long‐read sequencing revealed large haplotype blocks of ~1 Mb spanning the repeat expansion with crossover events across individuals from the Iowa and Swedish families (Fig. 1C). Assuming a correlated genealogy (as defined), the mutation arose 88.5 generations ago, with a 95% confidence interval (CI) of 23.7–400.6. Assuming that each generation is 25 years, this dates the mutation to ~2200 years ago (95% CI: 600 to 10,025 years).

Haplotype‐specific methylation calling around the *ZFHX3* GGC repeat expansion for individuals with SCA4 from Utah and Sweden showed hypermethylation around the repeat expansion site compared with the allele that does not harbor the repeat expansion (Supplementary Fig. S1). Of note, in those with homozygous wildtype alleles without the pathogenic repeat expansion, there was evidence of hypomethylation around the site of the non‐expanded short tandem repeat (Supplementary Fig. S1). This pattern of methylation suggests that the presence of the repeat expansion may have potential functional implications in gene or transcript expression in the disease state. Therefore, further characterization of the transcript structure and expression in disease within the brain would be invaluable to correlate these findings.

## 4 Discussion

In line with the common Swedish ancestry of all reported individuals affected by SCA4,^1^, ^2^, ^3^, ^4^, ^5^ our extended haplotype analysis in other families including those from Southern Sweden^3^ revealed a common single ~1 Mb haplotype harboring the pathogenic expansion. The presence of a founding haplotype of the *ZFHX3* GGC expansion demonstrates the phenomenon observed across other repeat expansion disorders including, but not limited to *C9orf72*,^15^ *NOTCH2NLC*,^16^, ^17^, ^18^ and *BEAN1* ^19^ that exhibit population‐specific differences in prevalence.

The *ZFHX3* GGC repeat expansion likely arose approximately 88.5 generations (~2220 years ago) (95% CI: 23.7–400.6 generations). This repeat‐containing haplotype arose much later than the *RFC1* repeat containing haplotype in CANVAS (cerebellar ataxia with neuropathy and bilateral vestibular areflexia syndrome), which is postulated to have arisen approximately 25,880 years ago (95% CI: 14,080–48,020).^20^ This may in part explain the higher prevalence of the *RFC1* expansion, which has a carrier frequency of 1 in 14.^21^ In comparison, the *C9orf72* hexanucleotide repeat expansion arose before, or just before the *ZFHX3* repeat containing haplotype and also has a Northern European founder (100.5 to 251 generations).^15^, ^22^

Although a recent study successfully detected the *ZFHX3* GGC repeat expansion through polymerase chain reaction (PCR)‐based methods from genomic DNA,^6^ most groups have experienced difficulties in detecting the *ZFHX3* repeat by PCR‐based methods due to its high GC content.^2^, ^3^, ^4^, ^5^ Thus, these findings support examination of the six rare SNVs tagging the expansion in screening for the pathogenic repeat‐containing haplotype. Furthermore, flanking sequences as well as interruptions that alter the repeat motif have been shown to have impact on the repeat stability and pathogenicity.^23^, ^24^ While we do not fully understand factors that moderate expansion of a short tandem repeat, a comprehensive knowledge of variants associated with the repeat‐containing haplotype structure has important implications when considering potential development of repeat‐stabilizing targeted therapy.

While SCA4 has been heralded as a novel polyglycine disorder given the GGC repeat expansion is the first to be described to reside in a coding exon,^7^ our findings of hypermethylation around the repeat expansion is intriguing. Hypermethylation has been appreciated in other GGC repeat expansion disorders such as in Fragile X syndrome, leading to transcriptional silencing of *FMR1*.^25^ However, in this case, the hypermethylation is within a coding exon of a gene, with previous evidence showing possible exon‐level DNA methylation may play a role in exon inclusion in alternative splicing; a trend observed generally within the last coding exons.^26^, ^27^

Of significance, ubiquitinated neuronal intranuclear inclusions were characterized within the post‐mortem brain tissue of the Swedish individuals with SCA4 studied here^3^ as well as in those from German families.^5^ While Figueroa and colleagues found that antibodies targeting ZFHX3 were detected in SCA4 brains and that there was an increase in ZFHX3 without concomitant increase in *ZFHX3* expression in patient‐derived fibroblasts but not controls, further understanding of the underlying pathogenesis remains to be elucidated.^5^ It is unknown whether the level of toxicity arises from toxic gain‐of‐function from the polyglycine‐containing protein or from a transcribed repeat‐containing RNA, or both. This is further complicated by the unknown significance of methylation around the GC‐rich repeat expansion.

Phased long‐read sequencing data from individuals from Utah, Iowa, and Southern Sweden has allowed detection of a common ancestral haplotype harboring the repeat expansion that is hypermethylated, providing further insight into not only the genetic architecture of SCA4, but also other repeat expansion disorders.

## Author Roles

(1) Research Project: A. Conceptualization, B. Methodology, C. Investigation, D. Data Curation, E. Formal Analysis; (2) Statistical Analysis: A. Design, B. Execution, C. Review and Critique; (3) Manuscript Preparation: A. Writing of the First Draft, B. Review and Critique; (4) Other: A. Funding Acquisition, B. Supervision.

Z.C.: 1A, 1B, 1C, 1D, 1E.

P.A.J.: 1B, 1C, 1D, 1E.

C.A.: 1B, 1C, 1D, 1E.

M.P.: 1B, 1C, 1D, 1E.

J.L.: 1B, 1C, 1D, 1E.

D.N.: 1C, 1D, 1E.

H.M.: 1C, 1D, 1E.

A.S.: 4A, 1C, 1D, 1E.

K.M.: 1C, 1D.

J.H.: 4A, 1C, 1D.

A.B.S.: 1C, 1D.

A.T.: 1C, 1D.

K.D.M.: 1C, 1D.

Y.‐H.F.: 1C, 1D.

M.E.: 1C, 1D.

J.L.‐M.: 1C, 1D.

I.N.: 1C, 1D.

A.W.: 1C, 1D.

L.J.P.: 1C, 1D.

C.B.: 4A, 1C, 1D.

E.K.G.: 1A, 1B, 1C, 1D, 1E, 4B.

P.S.: 1A, 1B, 1C, 1D, 4A, 4B.

M.R.: 1A, 1B, 1C, 1D, 4A, 4B.

H.H.: 1A, 1B, 1C, 1D, 4A, 4B.

## Supporting information


**Figure S1.** Haplotype‐specific methylation calling around the *ZFHX3* GGC repeat expansion for individuals with SCA4 from Utah (a) (Utah1: DNA was extracted from patient‐derived lymphoblastoid cell lines), (b) Sweden (Sweden1: DNA was extracted from post‐mortem brain hypothalamus), and (c) a control individual with no repeat expansion on either allele. DNA for this individual was derived from the HG002 cell line from Coriell (https://www.coriell.org/) as previously described.^13^ Deviation from the baseline methylation frequency and position of maximal difference in methylation between the two haplotypes represents the location of the repeat expansion. The vertical dashed line marks the starting position of the repeat expansion location. The haplotype containing the repeat expansion (orange line haplotype 2 in both Utah1 and Sweden1) shows higher methylation frequency around the site of the repeat expansion compared with the non‐repeat expansion haplotype. In the non‐expanded control, there is hypomethylation around the start site of the naturally occurring GGC short tandem repeat (n = 21 repeats). Methylated bases are represented in red on the reads. Unmethylated bases are shown in the blue sites. Visualization was created using modbamtools.^12^

## Data Availability

The data that support the findings of this study are available on request from the corresponding author. The data are not publicly available due to privacy or ethical restrictions.
