# Supplementary figures and images for: The ZFHX3 GGC Repeat Expansion Underlying Spinocerebellar Ataxia Type 4 has a Common Ancestral Founder
Source: Mov Disord. 2024 Dec 5;40(2):363–9. doi: 10.1002/mds.30077 (PMC11832790; doi:10.1002/mds.30077)

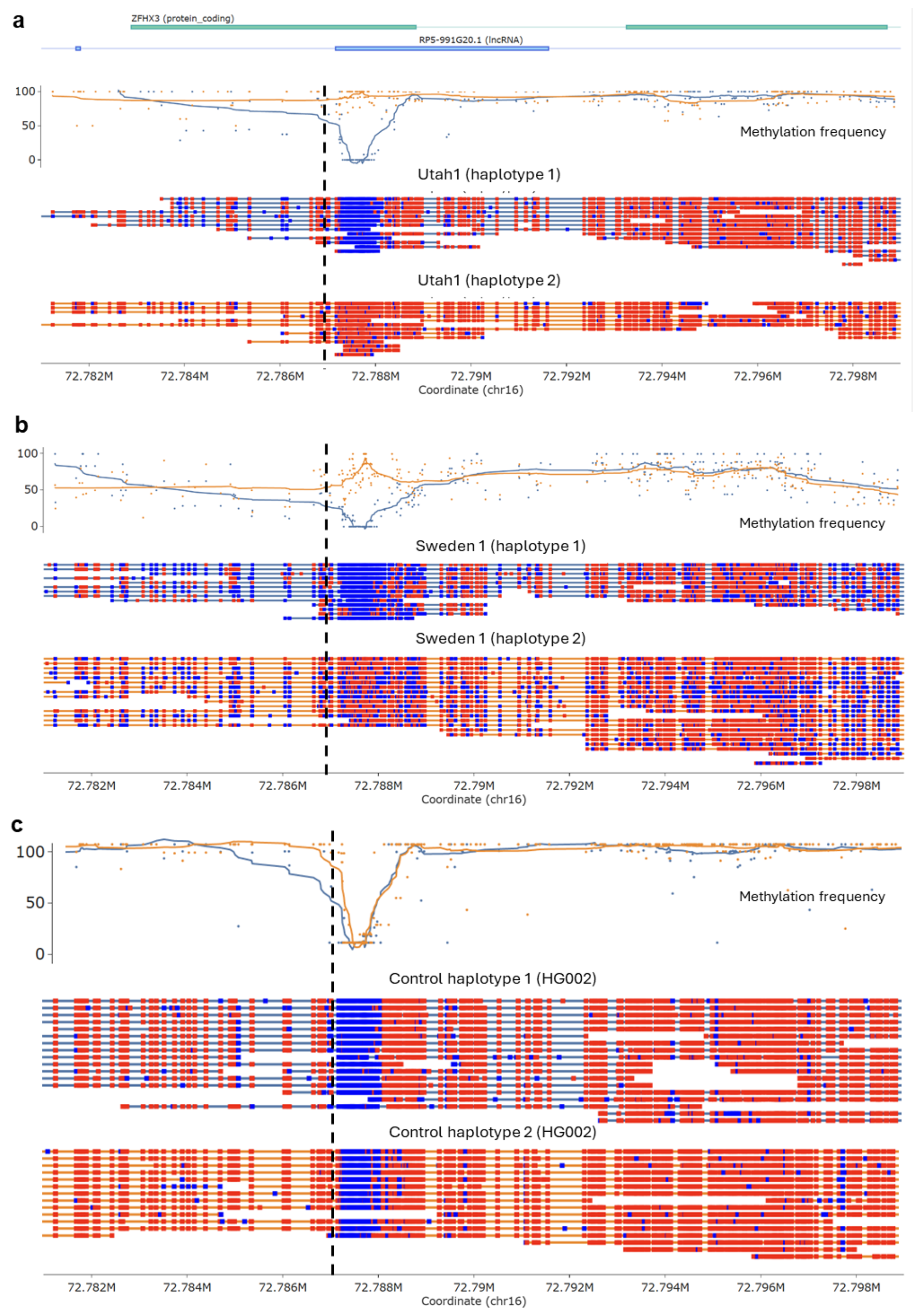

Supplement: Supplementary file 1 — Figure S1. Haplotype‐specific methylation calling around the ZFHX3 GGC repeat expansion for individuals with SCA4 from Utah (a) (Utah1: DNA was extracted from patient‐derived lymphoblastoid cell lines), (b) Sweden (Sweden1: DNA was extracted from post‐mortem brain hypothalamus), and (c) a control individual with no repeat expansion on either allele. DNA for this individual was derived from the HG002 cell line from Coriell (https://www.coriell.org/) as previously described. 13 Deviation from the baseline methylation frequency and position of maximal difference in methylation between the two haplotypes represents the location of the repeat expansion. The vertical dashed line marks the starting position of the repeat expansion location. The haplotype containing the repeat expansion (orange line haplotype 2 in both Utah1 and Sweden1) shows higher methylation frequency around the site of the repeat expansion compared with the non‐repeat expansion haplotype. In the non‐expanded control, there is hypomethylation around the start site of the naturally occurring GGC short tandem repeat (n = 21 repeats). Methylated bases are represented in red on the reads. Unmethylated bases are shown in the blue sites. Visualization was created using modbamtools. 12 [file MDS-40-363-s001.png]
